# Implementation process and acceptability of the electronic community health information system among community health workers in Kenya

**DOI:** 10.3389/frhs.2026.1818937

**Published:** 2026-06-11

**Authors:** Grace Njeri Muriithi, Robert Arasa, Theresia Mueni Mutetei

**Affiliations:** 1 Strathmore Business School, Strathmore University, Nairobi, Kenya; 2 School of Business and Economics, Machakos University, Machakos, Kenya; 3 Department of Business, Pan Africa Christian University, Nairobi, Kenya

**Keywords:** community health workers (CHWs), digital health, electronic community health information system (eCHIS), implementation process, mobile health, Normalization MeAsure Development (NoMAD), Normalization Process Theory (NPT), Technology Acceptance Model (TAM)

## Abstract

**Introduction:**

The electronic Community Health Information System (eCHIS) is a mobile health technology that was scaled nationally to community health workers (CHWs) in Kenya beginning in 2023. Since the rapid scale up, reports on eCHIS uptake show varying adoption rates. The overall purpose of this study was to assess the relationships between implementation process and acceptability of eCHIS by CHWs in Kenya.

**Methods:**

A cross-sectional study was conducted in 5 counties in Kenya with 310 CHWs (40 community health assistants and 270 community health promoters) selected through convenience stratified sampling. A structured questionnaire, developed by integrating the Technology Acceptance Model questionnaire and the Normalization MeAsure Development (NoMAD) tool, was administered by trained research assistants using Kobo Collect. The data was analyzed using descriptive statistics and structural equation modelling in STATA version 18.

**Results:**

Implementation process mechanisms were found to have positive and significant relationships with the acceptability of eCHIS. Specifically, cognitive participation had a significant and positive relationship with perceived usefulness of eCHIS (*β* = .2783, *p* < .05). Additionally, coherence (*β* = .3911, *p* < .001) and reflexive monitoring (*β* = .3272, *p* < .05) had significant and positive relationships with perceived ease of use. The study also revealed inadequate training, financial resources, and technical and management support as the key implementation barriers.

**Conclusions:**

Implementation process significantly influences the acceptability of eCHIS. It is therefore important for governments and other implementing partners to strengthen the eCHIS training programs, allocate sufficient financial and technical resources, and provide management support to the CHWs to enhance eCHIS adoption.

## 1 Introduction

Community health workers (CHWs) are increasingly recognized as an important human resource for health component for the achievement of universal health coverage (UHC) in low- and middle-income countries (1, 2). This is mainly due to the shortage of healthcare workers and the disproportionate distribution of healthcare workers in favour of urban settings (3). This has left rural and remote regions with inadequate supply of HCWs, a major factor contributing to regional disparities in health outcomes amongst populations. They are particularly instrumental in disease prevention and promotion as well as referral to health facilities and health education amongst their communities, thereby helping in strengthening primary health care (4).

Despite their significant contribution to the health sector, community health workers encounter many challenges during the execution of their work. These challenges include: low (and in many cases lack of) remuneration (both financial and non-financial) for their work, limited access to basic supplies needed to carry out their work, the need to travel long distances and cover wide geographical areas to meet their clients, use of manual tools, and lack of formal training on community health, among others (5–7). In trying to address the challenges facing the CHWs, many CHW programs and governments are implementing various measures including financial remuneration of the CHWs, non-financial incentives such as provision of transport for the CHWs, increased supervision, provision of formal training for the CHWs, and digitalization of the tools used by the CHWs.

For CHWs specifically, digital health technologies have been found to enhance efficiency of data collection in that CHWs can collect data from more clients within a shorter duration of time. DHTs have also enhanced the quality of the data collected by flagging potential errors during the data collection process. DHTs have also been found to improve service delivery, for instance, timely follow up of clients, efficient referral of clients to primary health care facilities, as well as better management of medicines and medical supplies (8–11). Effective supervision of the CHWs is also another benefit provided by the DHTs through performance dashboards (12).

While digital health technologies for community health workers have been in existence in Kenya for the past two decades, the major challenge experienced was that they were often donor-driven, fragmented and implemented in silos (13). To address these challenges, the national government began the national scale up of a mobile health technology for CHWs called the electronic Community Health information system (eCHIS). The eCHIS was modelled after the Smart Health Application developed by Living Goods and the Community Health Toolkit developed by Medic (14). The rollout of eCHIS in Kenya was supported by various policies including the Kenya Health Policy 2014–2030, the Kenya National eHealth Policy 2016–2030, the National Community Health Digitization Strategy 2020–2025, and the Digital Health Act 2023.

The eCHIS was first piloted in Kisumu County in 2021. Based on the feedback received during the pilot phase, the eCHIS was updated and the national scale-up was officially launched by the President of Kenya in August 2023. As of February 2024, more than fifty percent of the country's 47 counties had rolled out the eCHIS and trained their CHWs on its use (15). By mid 2025, all the counties had rolled out the eCHIS to their CHWs. Since the national scale up of eCHIS, anecdotal reports from implementing partners such as Living Goods (16) show mixed outcomes in terms of its adoption, defined as uptake and sustained use, with some CHWs reverting to the paper-based tools they used previously, citing technical and infrastructure-related challenges. Additionally, an empirical study conducted in two sub-counties of Migori County (17) found that the likelihood of adopting the eCHIS varied significantly between the two sub-counties, with CHWs in Nyatike sub-county having 14 times higher likelihood of adopting it than those in Awendo sub-county. It is therefore necessary to examine factors contributing to the differential adoption of eCHIS.

The Technology Acceptance Model (TAM) has been used extensively to identify factors that influence users’ intention to use or their actual usage of new technologies. The TAM argues that the actual usage of a new technology is influenced by the users’ intention to use the technology, which is in turn influenced by the users’ perceived usefulness and perceived ease of use of the technology (18–20). Perceived usefulness is defined as the belief that the new technology is more beneficial than the status quo, whereas perceived ease of use is defined as the belief that the new technology requires less effort to learn and use the technology (18–20). Together, these variables determine the extent to which a new technology will be accepted by the new users. The TAM has also been extended by adding other external variables to the model including health risk factors such as anxiety and fear; technology-related factors such as design, user experience, and aesthetics; social constructs such as social influence, social awareness, and subjective norms; digital literacy and perceptions such as self-efficacy, compatibility, computer anxiety, and attitude; and trust and privacy-related factors such as perceived risk, trust in government, and perceived privacy (21). Hence, a key gap in the Technology Acceptance Model literature is the lack of studies integrating implementation science models with the TAM to explain technology acceptance.

The application of implementation science theories in the field of digital health technologies has emerged as a strong framework for explaining facilitating factors and barriers to successful adoption of new DHTs. One notable theory is the Normalisation Process Theory (NPT) developed in 2009 by May and colleagues (22–24), who tried to explain how new innovations are introduced and become normalised into the daily routine of the users. The NPT states that implementation process works in four mechanisms namely: coherence (the process by which users make sense of new technology), cognitive participation (the process by which users engage with the new technology), collective action (the work the users have to do to make the new technology part of their daily routine of practice), and reflexive monitoring (the process by which feedback is collected to evaluate the performance of the new technology). The NPT therefore focuses on the work the users of a new technology must do both individually and collectively for the technologies to be successfully adopted (22–24), and as such, it also highlights organisation-related factors that influence technology adoption, and which have not been addressed in the TAM literature. Integrating the TAM model and the NPT theory would provide a stronger theoretical framework for analysing factors influencing the adoption of eCHIS in Kenya combining perceptions, individual effort, collective effort, and organisation-wide factors.

While the TAM model has been tested extensively in different contexts including developed and developing countries, and with both mainstream healthcare professionals and low-cadre health workers (25, 26), the NPT theory has significant context-related gaps. Majority of the studies applying the NPT have been conducted in developed countries, in formal healthcare organisations and facilities, and with the mainstream healthcare professionals (27–33). There is a dearth of evidence on the applicability of the NPT in developing countries, in community health systems and with low-cadre health workers such as community health workers working at the last mile. This study attempts to fill this evidence gap.

**Figure 1:**
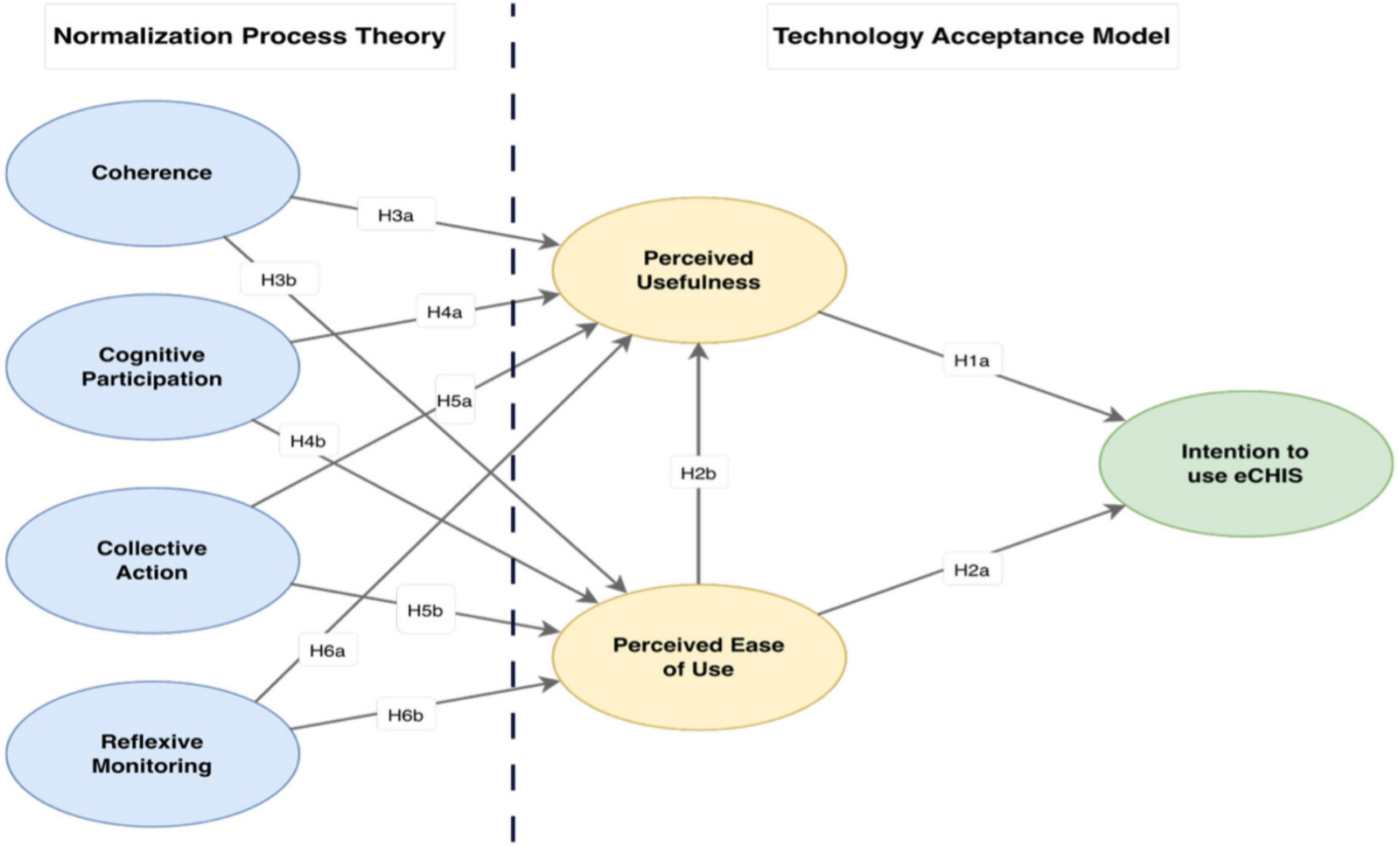
Conceptual framework for the study.

The main objective of this study is to integrate the Technology Acceptance Model with the Normalisation Process Theory to assess the direct relationships between implementation process mechanisms and acceptability of the electronic Community Health Information System in Kenya by community health workers. The study will test the following hypotheses (also shown in the conceptual framework in Figure 1):

H1a: Perceived usefulness has a significant and positive relationship with intention to use eCHIS in Kenya
H2a: Perceived ease of use has a significant and positive relationship with intention to use eCHIS in Kenya
H2b: Perceived ease of use has a significant and positive relationship with perceived usefulness of eCHIS in Kenya
H3a: Coherence has a significant and positive relationship with perceived usefulness of eCHIS in Kenya
H3b: Coherence has a significant and positive relationship with perceived ease of use of eCHIS in Kenya
H4a: Cognitive participation has a significant and positive relationship with perceived usefulness of eCHIS in Kenya
H4b: Cognitive participation has a significant and positive relationship with perceived ease of use of eCHIS in Kenya
H5a: Collective action has a significant and positive relationship with perceived usefulness of eCHIS in Kenya
H5b: Collective action has a significant and positive relationship with perceived ease of use of eCHIS in Kenya
H6a: Reflexive monitoring has a significant and positive relationship with perceived usefulness of eCHIS in Kenya
H6b: Reflexive monitoring has a significant and positive relationship with perceived ease of use of eCHIS in Kenya

## 2 Methods

### 2.1 Study design

The study adopted a quantitative cross-sectional research design in which data was collected from community health workers beginning in July 2024 and concluding in November 2024. The cross-sectional design was appropriate to this study because the eCHIS was still in the early phases of national rollout. It is therefore suited to assess whether and how adoption of eCHIS varies by the different demographic groups of the respondents. Cross-sectional survey design is also appropriate for testing relationships between the study's constructs and subsequently confirming or rejecting the research hypotheses (34).

### 2.2 Study population

The study population included approximately 110,250 community health workers in the 47 counties of Kenya. CHWs are of two main cadres namely community health promoters (CHPs), and their supervisors the community health assistants (CHAs). The CHPs carry out the day-to-day work of registering households in their catchment areas, collecting data, making follow-up visits to their clients, referring clients to the nearest health facilities, disease prevention, and disease surveillance. The CHAs on the other hand supervise the CHPs and escalate their issues to the county focal persons for redress.

### 2.3 Study participants

A sample size of 399 was considered adequate based on the population size and 5 percent margin. The sample size was determined by the Yamane formula (35):

$$n=\frac{N}{1+N\propto^{2}}$$ (1)

Where *n* is the sample size, *N* is the population size, and *∝* is the error, in this case 0.05.

Due to resource constraints, the final sample of the study included 310 community health workers (representing 78 percent of the ideal sample size) drawn from the five counties as follows: Nairobi (60 participants), Kisumu (70 participants), Migori (60 participants), Machakos (60 participants) and Isiolo (60 participants). The respondents were selected using convenience stratified sampling technique (36). To start with, the county community health focal persons (in Isiolo, Kisumu, Machakos and Migori counties) mapped the rural and urban community health units. Isiolo, Machakos and Migori counties are predominantly rural counties. However, Isiolo County is a vastly and sparsely populated county hence only 2 rural CHUs and 7 urban CHUs were selected to take part in the study due to budget limitations to cover long distances and pay for the accommodation of the research assistant. In Machakos County, 7 rural and 4 urban CHUs were selected for the study, whereas in Migori County, 9 rural CHUs and 3 urban CHUs were selected for the study. Kisumu County is predominantly an urban county, hence 5 rural CHUs and 8 urban CHUs were selected to participate in the study. Nairobi County is purely urban but with a large urban poor population. The CHUs selected in Nairobi County were those located in the informal settlements in 3 sub-counties. A random sample of between 4 and 6 CHWs were selected from each CHU, depending on the number of CHWs in each CHU. The recruitment of the CHWs in each CHU was done with the help of the Community Health Assistants, who head the CHUs. The recruited participants were then contacted by the respective research assistants who then informed them of the study, the study's objectives, and the nature of their participation, and their sought their permission to participate in the study. An introduction letter and informed consent form was shared with the recruited participants informing them of ethical issues such as confidentiality and privacy of the data collected, as well as voluntary participation.

### 2.4 Data collection

The study collected quantitative primary data from the respondents using a structured survey questionnaire (Supplementary material I). The questionnaire was developed and adapted from both the TAM questionnaire and the Normalization MeAsure Development (NoMAD) questionnaire which was developed by May et al. based on the NPT theory (36, 37). Section A of the questionnaire contained background information about the respondents (such as gender, age, experience as CHWs, cadre of CHWs, and experience with eCHIS and other mobile health technologies). Section B contained the measurement items for the TAM model followed by measurement items for the NPT theory, organized by their different constructs (intention to use eCHIS, perceived usefulness, perceived ease of use, coherence, cognitive participation, collective action, and lastly, reflexive monitoring). In section B, the measurement indicators were measured on a 5-point Likert scale measuring the degree to which the respondents agreed with each of the statements (the scale ranged from 1 = strongly disagree to 5 = strongly agree). The use of the 5-point Likert scale for the constructs was consistent with previous studies that applied the TAM and NoMAD questionnaires (27, 32, 33). Adaptation of the questionnaire was done by replacing the word “technology” in the TAM questionnaire and the word “intervention” in the NPT questionnaire with the word “eCHIS”. Sample statements in the questionnaire include: “I intend to continue using eCHIS”, “I am able to understand how eCHIS affects the nature of my work”, and “It is easy to integrate the eCHIS into existing work”.

Data collection was conducted by trained research assistants using the *Kobo Collect* mobile application. Prior to the actual data collection, a pretest of the questionnaire was done with ten randomly selected CHWs from the 5 counties. The rationale for the pretest was to ensure that the questions in the questionnaire could easily be answered by the respondents with minimal difficulties and ambiguity. The pretest revealed ambiguity with the meaning of measurement item Collective Action_2 “The eCHIS disrupts working relationships.” Hence, a meeting with the research assistants was held to further clarify the meaning of the measurement item so that all the research assistants were aligned with its meaning.

### 2.5 Data analysis

Data was analyzed using descriptive statistics and structural equation modelling (SEM). The descriptive statistics (mean, median, frequency and percentage) were used for the demographic variables as well as for the scores of the study's constructs. Prior to running the SEM analysis, the assumptions of sample adequacy, model identification, normality, and no multicollinearity were tested. Sample adequacy was determined by the rule of thumb of between 5 and 10 cases per indicator (38). The model had 37 indicators hence a minimum sample of 185 was required to attain sample adequacy. Other guidelines provided in the SEM literature include a minimum sample of 200 cases (39), which this study attained.

Model identification was assessed using the goodness-of-fit post-estimation analysis. For a SEM model to run, it must be overidentified, that is, the degrees of freedom should be greater than zero (39). The assumption of normality was assessed using the joint skewness and kurtosis test, which tests the null hypothesis of normal distribution. If the *p*-value is less than 0.05, it means that the null hypothesis is rejected and that the variable is not normally distributed. On the other hand, a *p*-value greater than 0.05 means that the variable is normally distributed. The assumption of no multicollinearity was tested using variance inflation factor (VIF) and tolerance (39). Variance inflation factor values that are less than the threshold of 10 imply that there is no multicollinearity. Similarly, tolerance values greater than 0.1 imply no multicollinearity.

**Figure 2:**
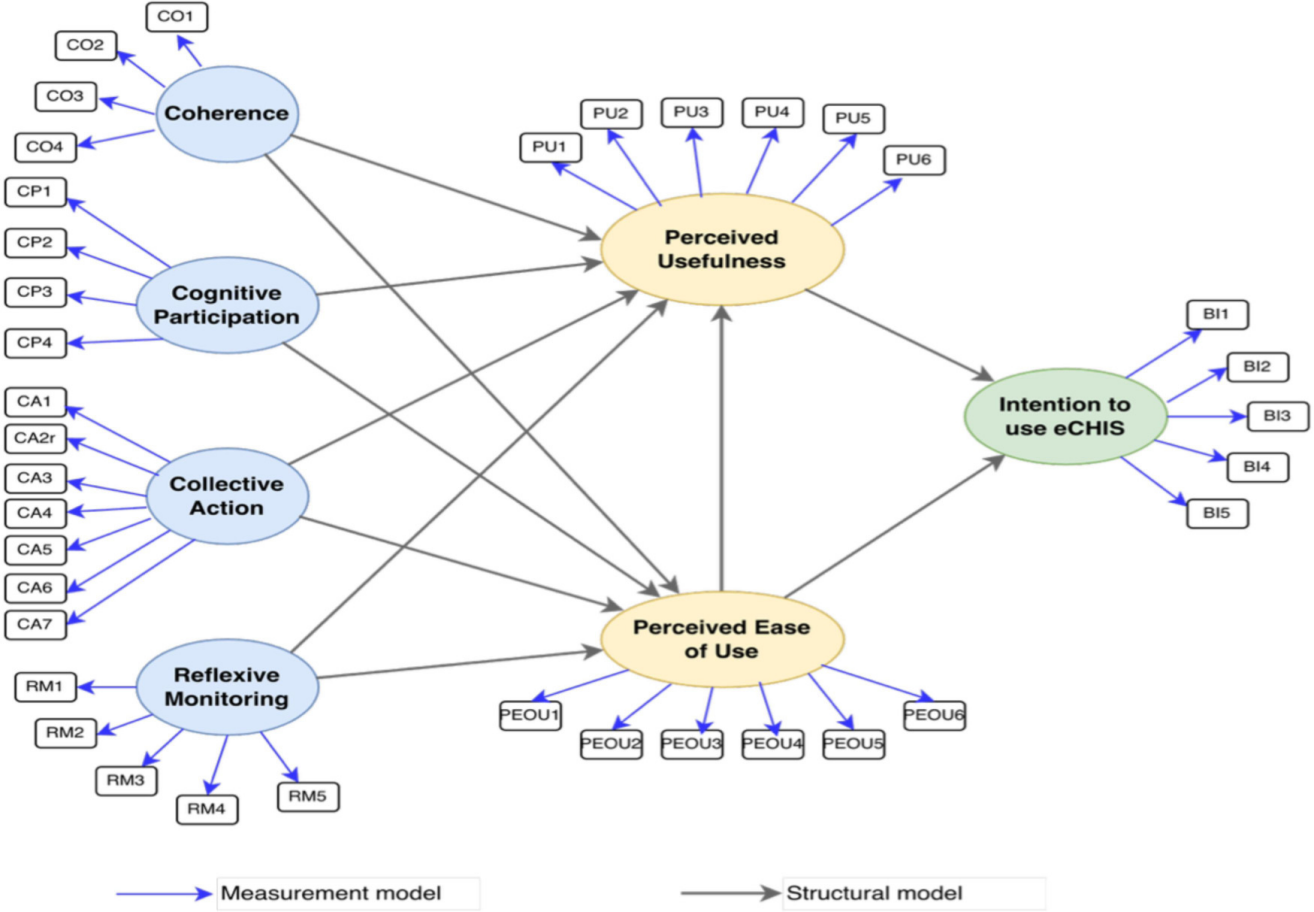
Structural equation model for the study.

Structural equation modelling was used to test the relationships between the NPT constructs and the TAM constructs. SEM was appropriate for this study because of the latent nature of the variables under investigation, which cannot be directly observed but are instead measured using observed measurement items (39). Structural equation modelling entails analyzing two models namely the measurement model and the structural model using confirmatory factor analysis (CFA) and path analysis, respectively (Figure 2). The measurement model shows how the constructs are measured and how reliable and valid they are, whereas the structural model shows the strength and direction of the relationships between the constructs. The reliability of the constructs was tested using Cronbach's alpha, whose threshold is 0.70. Construct validity was tested by both convergent and divergent validity using the Average Variance Extracted, whose threshold is 0.50, and the Fornell-Larcker Criterion, which states that the square root of a construct's AVE should be greater than its correlations with other constructs (39). Additionally, the goodness-of-fit of the models was tested using a combination of the Chi-square test and fit indices such as Goodness of Fit Index, Root Mean Square of Error Approximation (RMSEA), Standardized Root Mean Square Residual (SRMR), Comparative Fit Index (CFI) and Incremental Fit Index (IFI) (38). All analyses were conducted in STATA version 18.

## 3 Results

### 3.1 Socio-demographic characteristics of respondents

The socio-demographic profile of the respondents is presented in Table 1. Of the 310 respondents, the majority (270) were community health promoters and only 40 were community health assistants. There were more females (222) than males (88), and more urban respondents (185) than rural respondents (125). Majority of the respondents (253) owned a smartphone prior to the introduction of eCHIS, while only 18 percent did not own a smartphone. Half of the respondents had used an mHealth technology prior to the introduction of eCHIS, while the other half had no prior experience with an mHealth technology. The mean age of the respondents was 41 years, with the youngest respondent aged 23 years and the oldest respondent aged 78 years.

**Table 1:** Socio-demographic characteristics of respondents.

Variable	Count (percent)
Age:	
Mean age	41.2
Age range	23–78
Age group:	
40 and below	167 (53.9)
41 and above	143 (46.1)
Gender:	
Male	88 (28.4)
Female	222 (71.6)
Education level:	
Primary	66 (21.3)
Secondary	170 (54.8)
Tertiary	74 (23.9)
Cadre of community health workers:	
Community health assistants	40 (12.9)
Community health promoters	270 (87.1)
Location:	
Rural	125 (40.3)
Urban	185 (59.7)
Smartphone ownership prior to eCHIS:	
No	57 (18.4)
Yes	253 (81.6)
Prior use of any other mHealth tool:	
No	155 (50.0)
Yes	155 (50.0)
Preference for eCHIS or paper-based tools:	
eCHIS	3 (0.97)
Paper-based tools	307 (99.03)

### 3.2 Level of acceptability of eCHIS among the respondents

**Figure 3:**
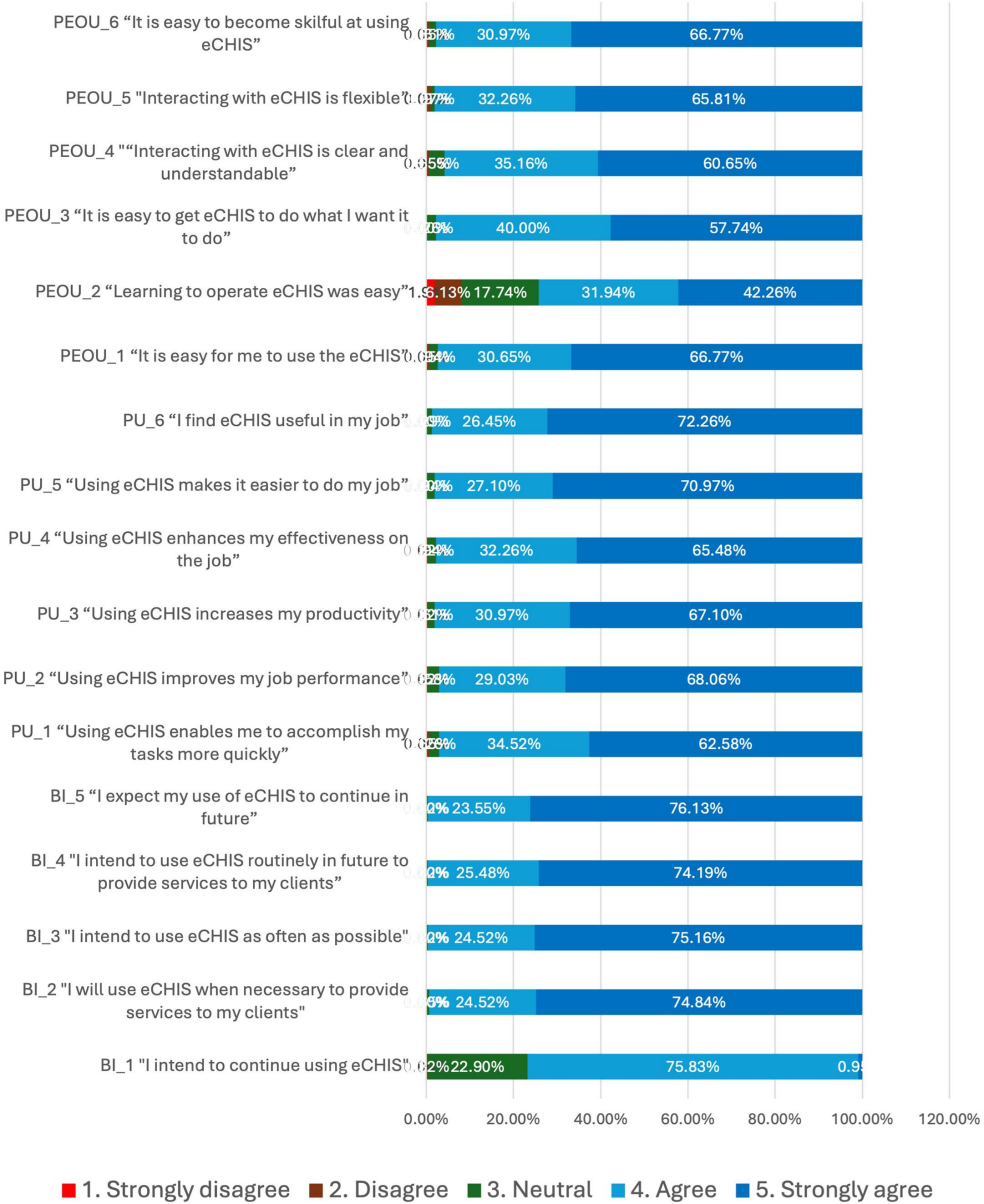
Percentage scores on Technology Acceptance Model items.

The level of acceptability of eCHIS was measured using three TAM variables: perceived ease of use, perceived usefulness, and intention to continue using eCHIS. The percentage scores on each of the TAM items are presented in Figure 3.

#### 3.2.1 Intention to continue using eCHIS

The intention to continue using eCHIS was measured using five items (BI_1 to BI_5 in Figure 3). Seventy-seven percent of the respondents either agreed or strongly agreed with the item “I intend to continue using eCHIS” whereas 23 percent were neutral about it. On the other hand, more than 99 percent of the respondents either agreed or strongly agreed with the remaining four items measuring intention to continue using eCHIS.

#### 3.2.2 Perceived usefulness of eCHIS

The perceived usefulness construct was measured using six items (PU_1 to PU_6 in Figure 3). More than 97 percent of the respondents either agreed or strongly agreed with all the six items measuring perceived usefulness. Hence for the majority of the respondents, the eCHIS was perceived as enabling them to accomplish their tasks more quickly (PU_1), improving their job performance (PU_2), increasing their productivity (PU_3), enhancing their effectiveness on their job (PU_4), and making their job easier (PU_5). The eCHIS was also perceived as useful in their job (PU_6).

#### 3.2.3 Perceived ease of use of eCHIS

The perceived ease of use construct was also measured using six items (PEOU_1 to PEOU_6 in Figure 3). More than 97 percent of the respondents found the eCHIS easy to use (PEOU_1), easy to get it to do what they want (PEOU_3), flexible (PEOU_5) and to become skilful at using eCHIS (PEOU_6). About 96 percent of the respondents also found that interacting with eCHIS was clear and understandable (PEOU_4). On the other hand, learning to use eCHIS (PEOU_2) at the beginning was only easy for about 74 percent of the respondents, with 8 percent of the respondents either disagreeing or strongly disagreeing with the statement, with an additional 18 percent being neutral about it. The implication is that the learning curve was steep for about 26 percent of the respondents.

### 3.3 Implementation process mechanisms

**Figure 4:**
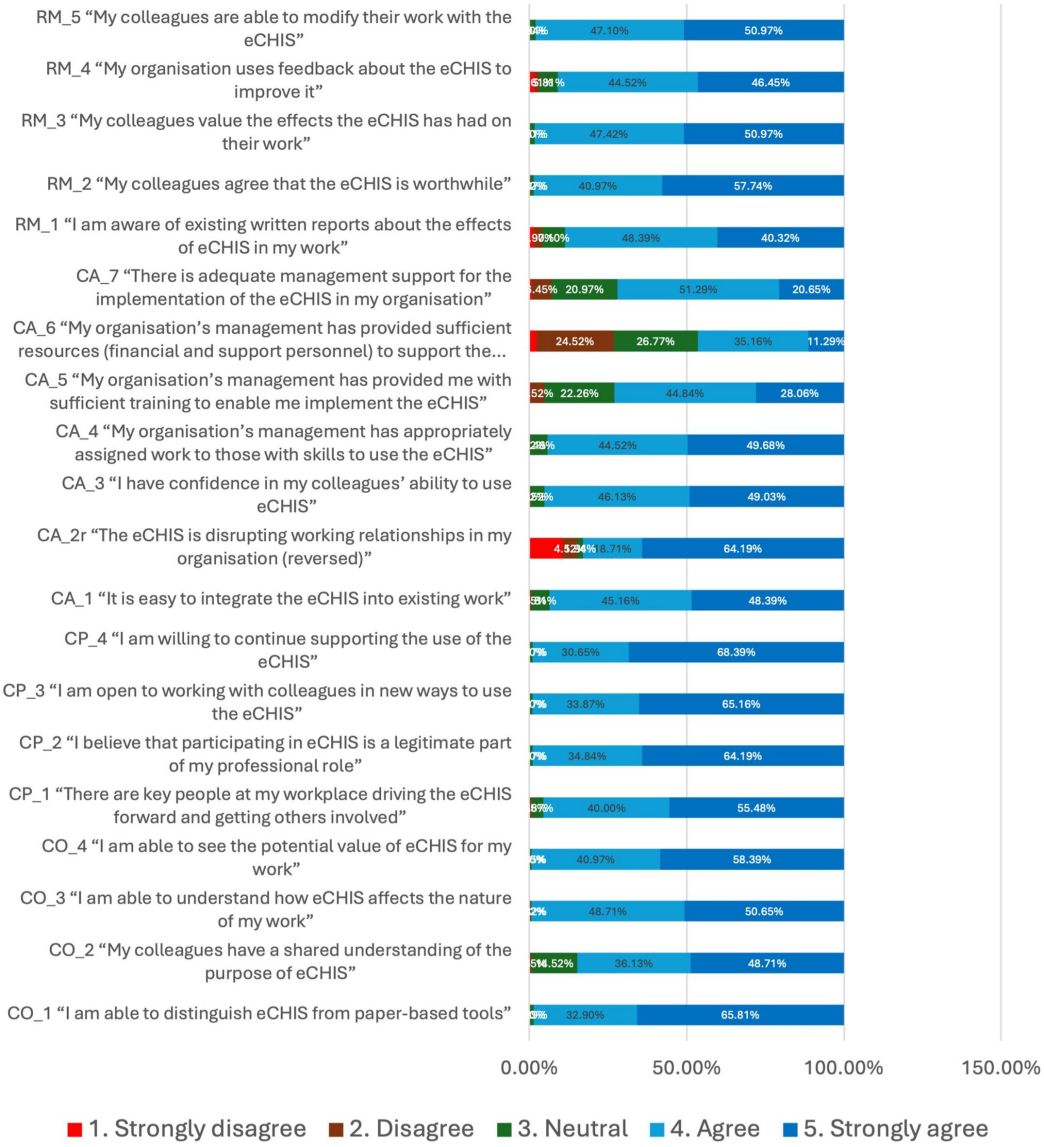
Percentage scores on normalisation process theory items.

The implementation process construct was based on the four NPT constructs namely coherence, cognitive participation, collective action, and reflexive monitoring. The percentage scores on each of the NPT items are presented in Figure 4.

#### 3.3.1 Coherence

Coherence refers to the sensemaking of the new technology by the users, particularly in comparison to the status quo or prior ways of working. It considers the need for the new technology given the working environment, the value added and the benefits the users derive from using the new technology compared to the old ways of working (27–31). The coherence construct was measured using four items (CO_1 to CO_4 in Figure 4). More than 98 percent of the respondents either agreed or strongly agreed that they were able to distinguish eCHIS from paper-based tools (CO_1), they understood how eCHIS affected the nature of their work (CO_3), and they saw the potential value of eCHIS for their work (CO_4). On the other hand, fewer respondents (85 percent) agreed or strongly agreed that their colleagues had a shared understanding of the purpose of eCHIS (CO_2), while 15 percent were neutral about the statement.

#### 3.3.2 Cognitive participation

Cognitive participation refers to the buy-in and engagement of the users with the new technology (27, 28, 30), presence of champions of the new technology who advocate for and promote its use among their colleagues (29), the manner in which the users come to learn how to use the new technology including the skills required to use it (29, 31), matters to do with teamwork and communication strategies about the new technology (34), and the willingness of the users to continue supporting the new technology. The cognitive participation construct was also measured using four items (CP_1 to CP_4). About 95 percent of the respondents believed that there are champions of eCHIS who drive it forward and get other CHWs involved (CP_1). Additionally, more than 99 percent of the respondents either agreed or strongly agreed that participating in eCHIS was a legitimate part of their professional role (CP_2), they were open to working with their colleagues in new ways to use eCHIS (CP_3), and were willing to continue supporting the use of eCHIS (CP_4).

#### 3.3.3 Collective action

Collective action entails the effort put in by the users both individually and collaboratively to support the implementation of the new technology. It also entails the provision of adequate training for the users, management support, and provision of adequate resources to ensure that the implementation of the new technology is successful (27–30). The collective action construct was measured using seven items (CA_1 to CA_7). More than 93 percent of the respondents either agreed or strongly agreed that it was easy to integrate eCHIS into existing work (CA_1), while more than 94 percent of the respondents either agreed or strongly agreed that they had confidence in their colleagues’ ability to use eCHIS (CA_3) and that work had been appropriately assigned to those with skills to use eCHIS (CA_4). More than 82 percent of the respondents agreed or strongly agreed that eCHIS was disrupting working relationships. On the hand, fewer respondents (about 72 percent) agreed or strongly agreed that management had provided them with sufficient training (CA_5) and management support (CA_7) to effectively implement eCHIS. Even fewer respondents (46 percent) agreed or strongly agreed that management had provided them with sufficient resources (financial and technical support) to implement the eCHIS (CA_6).

#### 3.3.4 Reflexive monitoring

Reflexive monitoring refers to the appraisal of the new technology by the users and how the technology affects their work (27, 29). It also entails efforts made to improve the technology and work practices based on the feedback provided by the users (28, 30). The reflexive monitoring construct was measured using five items. More than 98 percent of the respondents agreed or strongly agreed that their colleagues found the eCHIS worthwhile (RM_2), their colleagues valued the effects the eCHIS had on their work (RM_3) and were able to modify their work with the eCHIS (RM_5). However, fewer respondents (88 percent) were aware if written reports about the effects of eCHIS (RM_1), and about 90 percent agreed or strongly agreed that their organisation uses feedback about eCHIS to improve it (RM_4).

### 3.4 Assumptions of structural equation modelling

#### 3.4.1 Normality assumption

The study found only one construct – collective action – to be normally distributed (Chi^2^(2) = 2.65, *p* > 0.05). The other constructs did not meet the normality assumption: intention to use eCHIS (Chi^2^(2) = 46.34, *p* < 0.05), perceived usefulness (Chi^2^(2) = 32.94, *p* < 0.05), perceived ease of use (Chi^2^(2) = 38.45, *p* < 0.05), coherence (Chi^2^(2) = 124.71, *p* < 0.05), cognitive participation (Chi^2^(2) = 32.27, *p* < 0.05), and reflexive monitoring (Chi^2^(2) = 57.68, *p* < 0.05). Due to the violation of the assumption of normality for majority of the constructs, the study utilised maximum likelihood with bootstrapping to estimate the SEM model. This technique is recommended when data is not normally distributed because it ensures the robustness of the standard errors and reliability of the fit indices (39).

#### 3.4.2 No multicollinearity assumption

We assessed if there was multicollinearity between the outcome construct (intention to use eCHIS) and the other constructs by analysing the variance inflation factors and tolerance values. The VIF values were perceived usefulness (2.16), perceived ease of use (2.13), cognitive participation (2.09), coherence (1.99), reflexive monitoring (1.98), and collective action (1.11), all of which were below the threshold of 10. On the other hand, the tolerance values were perceived usefulness (0.463), perceived ease of use (0.469), cognitive participation (0.478), coherence (0.501), reflexive monitoring (0.504), and collective action (0.900), all of which were greater than 0.1. Based on the VIF and tolerance values, the assumption of no multicollinearity was achieved.

#### 3.4.3 Model identification

We checked for model identification post-SEM estimation by assessing the degrees of freedom produced for the goodness-of-fit measures. The degrees of freedom were greater than zero hence the model was over-identified (the known parameters were greater than the unknown parameters), allowing the model to be estimated (39).

### 3.5 Reliability and validity of the study's instruments

Reliability and validity of the constructs were done in Stata using the *validscale* and *condisc* commands, which provide information on Cronbach alpha, correlations between the measurement items and the constructs, average variance extracted and squared correlations between the constructs (40, 41). As such, the two commands flag which items cause reliability and validity issues. Reliability in this study was assessed using the Cronbach's alpha, whose minimum threshold is 0.70. On the other hand, the study assessed construct validity of the measurement items and constructs using convergent and divergent validity. Convergent validity is best assessed using average variance extracted (AVE), which should be greater than 0.50 (38). Divergent validity was assessed by comparing the AVE values with the squared correlations among the latent variables. It is achieved if the AVE values are greater than or equal to the squared correlations values for each construct. When all the items were included in the analysis, the reliability failed to meet the minimum threshold for Cronbach alpha (0.70) for collective action (0.57). Similarly, the convergent validity of both collective action (0.266) and reflexive monitoring (0.469) failed to meet the AVE threshold of 0.50, implying that the two constructs explained less than half of the variances in their measurement indicators. Similarly, divergent validity failed to establish in five of the constructs, with the exception of intention to use eCHIS and perceived usefulness.

This improved only after five items from collective action (CA_1, CA_2, CA_3, CA_4 and CA_6) and one item from reflexive monitoring (RM_4) were removed. These items were flagged during the analysis. The removal of the items was done sequentially in the following order: CA_2r, CA_6, CA_1, CA_3, CA_4, and lastly RM_4). The reliability met the minimum threshold of 0.07 after CA_2r and CA_6 were dropped. Despite achieving reliability of all the constructs, the convergent and divergent validity of collective action was not achieved. This informed the removal of the remaining items based on their cross-loading with other constructs until both reliability and validity were established (Tables 2–4). Validity and reliability issues with the NPT questionnaire have also been found in other studies (42–45). In the study conducted in Sweden (42), both the collective action and reflexive monitoring constructs had validity issues forcing the authors to drop items CA_2 and CA_3 and RM_4. Freund et al. (43) removed the item RM_1 from their study whereas (44, 45) suggested removing the item CA_2. The implication is that the reliability and validity of the NPT questionnaire vary from one context to another.

**Table 2:** Factor loadings, reliability and validity of constructs (with all items included).

Construct	Items	Factor loadings	Cronbach's alpha	AVE	Squared correlations among constructs						
					BI	PU	PEOU	CO	CP	CA	RM
BI	BI-1	.8678	0.95	0.809	1.000						
	BI-2	.8838									
	BI-3	.9354									
	BI-4	.9187									
	BI-5	.8905									
PU	PU-1	.7782	0.93	0.702	0.459	1.000					
	PU-2	.8430									
	PU-3	.8391									
	PU-4	.7730									
	PU-5	.9025									
	PU-6	.8841									
PEOU	PEOU-1	.7919	0.91	0.642	0.416	0.510	1.000				
	PEOU-2	.5220									
	PEOU-3	.8283									
	PEOU-4	.8836									
	PEOU-5	.8835									
	PEOU-6	.8395									
CO	CO-1	.5984	0.83	0.551	0.252	0.401	0.468	1.000			
	CO-2	.6797									
	CO-3	.7647									
	CO-4	.8928									
CP	CP-1	.5755	0.85	0.599	0.391	0.473	0.369	0.463	1.000		
	CP-2	.8274									
	CP-3	.8528									
	CP-4	.8085									
CA	CA-1	.6005	0.57	0.266	0.198	0.376	0.465	0.644	0.486	1.000	
	CA-2r	−.3857									
	CA-3	.8110									
	CA-4	.5661									
	CA-5	.4704									
	CA-6	−.1756									
	CA-7	.3458									
RM	RM-1	.5628	0.80	0.469	0.326	0.471	0.442	0.513	0.640	0.572	1.000
	RM-2	.8004									
	RM-3	.8166									
	RM-4	.4180									
	RM-5	.7402									

**Table 3:** Factor loadings, reliability and validity of constructs (after dropping CA_2r and CA_6).

Construct	Items	Factor loadings	Cronbach's alpha	AVE	Squared correlations among constructs						
					BI	PU	PEOU	CO	CP	CA	RM
BI	BI-1	.8678	0.95	0.809	1.000						
	BI-2	.8838									
	BI-3	.9354									
	BI-4	.9187									
	BI-5	.8905									
PU	PU-1	.7782	0.93	0.702	0.459	1.000					
	PU-2	.8429									
	PU-3	.8391									
	PU-4	.7729									
	PU-5	.9025									
	PU-6	.8842									
PEOU	PEOU-1	.7921	0.91	0.642	0.416	0.510	1.000				
	PEOU-2	.5215									
	PEOU-3	.8281									
	PEOU-4	.8839									
	PEOU-5	.8836									
	PEOU-6	.8394									
CO	CO-1	.5982	0.83	0.551	0.252	0.401	0.468	1.000			
	CO-2	.6788									
	CO-3	.7656									
	CO-4	.8928									
CP	CP-1	.5752	0.85	0.599	0.391	0.473	0.369	0.463	1.000		
	CP-2	.8276									
	CP-3	.8530									
	CP-4	.8082									
CA	CA-1	.6000	0.74	0.348	0.176	0.362	0.440	0.627	0.449	1.000	
	CA-3	.8040									
	CA-4	.5747									
	CA-5	.5035									
	CA-7	.3843									
RM	RM-1	.5628	0.80	0.469	0.326	0.471	0.442	0.513	0.640	0.572	1.000
	RM-2	.8004									
	RM-3	.8166									
	RM-4	.4180									
	RM-5	.7402									

**Table 4:** Factor loadings, reliability and validity of constructs (after dropping CA_1, CA_2r, CA_3, CA_4, CA_6 and RM_4).

Construct	Items	Factor loadings	Cronbach's alpha	AVE	Squared correlations among constructs						
					BI	PU	PEOU	CO	CP	CA	RM
BI	BI-1	.8678	0.95	0.809	1.000						
	BI-2	.8836									
	BI-3	.9353									
	BI-4	.9190									
	BI-5	.8904									
PU	PU-1	.7781	0.93	0.702	0.459	1.000					
	PU-2	.8433									
	PU-3	.8393									
	PU-4	.7732									
	PU-5	.9022									
	PU-6	.8839									
PEOU	PEOU-1	.7917	0.91	0.642	0.416	0.510	1.000				
	PEOU-2	.5195									
	PEOU-3	.8264									
	PEOU-4	.8847									
	PEOU-5	.8852									
	PEOU-6	.8390									
CO	CO-1	.6029	0.83	0.550	0.253	0.401	0.466	1.000			
	CO-2	.6703									
	CO-3	.7646									
	CO-4	.8964									
CP	CP-1	.5677	0.85	0.599	0.392	0.472	0.369	0.464	1.000		
	CP-2	.8301									
	CP-3	.8499									
	CP-4	.8128									
CA	CA-5	.9024	0.74	0.642	0.003	0.035	0.068	0.172	0.021	1.000	
	CA-7	.6846									
RM	RM-1	.5628	0.80	0.538	0.331	0.480	0.456	0.532	0.468	0.081	1.000
	RM-2	.8004									
	RM-3	.8166									
	RM-5	.7402									

In addition to testing the reliability and validity of the instruments, confirmatory factor analysis (CFA) assessed how well the measurement items were explained by their respective constructs. The CFA was run in several rounds: the first one with all the items included in the model, and subsequent rounds with the removal of the five items from collective action and one item from reflexive monitoring done sequentially. The goodness-of-fit of each of the models improved, with the final model having an acceptable fit: RMSEA (0.076), CFI (0.903), TLI (0.892), and SRMR (0.057).

### 3.6 Direct relationships between implementation process and acceptability of eCHIS

Following the results from the reliability and validity tests, the final structural model excluded the 6 items that were found not to meet the reliability and validity thresholds. Bootstrapping technique with 200 replicates was used to test the structural model and the hypothesised relationships shown in Figure 1. The structural model had a good fit to the data: Chi2/df = 2.77, RMSEA = 0.076, SRMR = 0.057, CFI = 0.903, and TLI = 0.892. The model explained 51.8 percent of variation in intention to use eCHIS, 62.6 percent of variation in perceived usefulness of eCHIS, and 53.7 percent of variation in perceived ease of use of eCHIS, showing a good explanatory power. The results of the bootstrapped standardized coefficients measuring the hypothesised relationships between the constructs are presented in Table 5.

**Table 5:** Results of the hypothesized relationships.

Hypothesis	Relationships	Coefficients)	*p* value	Results
H1a	PU 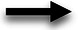 BI (+)	.4549***	0.000	Supported
H2a	PEOU 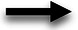 BI (+)	.3208***	0.000	Supported
H2b	PEOU 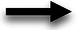 PU (+)	.3934***	0.000	Supported
H3a	CO 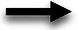 PU (+)	.0716	0.463	Not supported
H4a	CP 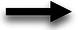 PU (+)	.2783*	0.045	Supported
H5a	CA 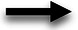 PU (+)	−.0332	0.457	Not supported
H6a	RM 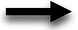 PU (+)	.1657	0.169	Not supported
H3b	CO 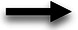 PEOU (+)	.3911***	0.000	Supported
H4b	CP 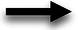 PEOU (+)	.0745	0.541	Not supported
H5b	CA 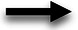 PEOU (+)	−.0076	0.890	Not supported
H6b	RM 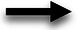 PEOU (+)	.3272*	0.031	Supported

*p < .05.

***p < .001.

The results show that perceived usefulness (*β* = .4549, *p* < .001) and perceived ease of use (*β* = .3208, *p* < .001) had significant and positive relationships with intention to use eCHIS. The relationship between perceived usefulness and intention to use eCHIS was stronger than the relationship between perceived ease of use and intention to use eCHIS. The positive relationships imply that the higher the scores on perceived usefulness and perceived ease of use, the higher the scores on intention to use eCHIS. Hence, the intention to use eCHIS would increase if the benefits derived from using eCHIS were perceived by the CHWs to be more than the paper-based tools, and if the effort required to learn and use eCHIS was not much.

Second, perceived ease of use (*β* = .3934, *p* < .001) and cognitive participation (*β* = .2783, *p* < .05) were found to have positive and significant relationships with perceived usefulness. The implication is that the higher the scores on perceived ease of use and cognitive participation, the higher the scores on perceived usefulness. Hence, CHWs would perceive the eCHIS to be more beneficial to them if they required less effort to learn and use it, and if they were actively engaged in its implementation.

Lastly, coherence (*β*=.3911, *p* < .001) and reflexive monitoring (*β*=.3272, *p* < .05) were found to have significant and positive relationships with perceived ease of use. The implication is that the higher the scores on coherence and reflexive monitoring, the higher the scores on perceived ease of use. Hence, the CHWs would find it easier to use eCHIS if they understood the purpose and effect of the eCHIS on their work, and if feedback was collected and used to improve not only eCHIS but also their work.

## 4 Discussion

The study provided evidence of significant and positive relationships between implementation process mechanisms and acceptability of the electronic community health information system in Kenya. The study supported the Technology Acceptance Model in that it found positive and significant relationships between perceived usefulness and intention to use eCHIS, as well as between perceived ease of use and intention to use eCHIS, which is consistent with other studies examining the TAM relationships (10, 46–53). While the study also supports other studies (10, 46–52) that found a significant and positive relationship between perceived ease of use and perceived usefulness, a few other studies found no such relationship (54). In Klingberg et al.'s study (54), the authors explained that almost all the participants in their study owned a smartphone and had already used it for work purposes including burn consultations, hence ease of use of the technology was not an issue to them.

High scores on perceived usefulness, perceived ease of use and intention to use constructs can be explained by several factors. To begin with, the eCHIS was preferred by more than 99 percent of the respondents in this study as compared to the paper-based tools used previously. This supports the perceived added benefits of eCHIS over the paper-based tools. Additionally, the smartphone ownership among the study's sample was high (82 percent), while half of the respondents had prior experience with other mobile health technologies before the eCHIS was rolled out. The high smartphone ownership and previous experience with mHealth technologies could explain the high levels of perceived usefulness and perceived ease of use of the eCHIS among the respondents.

In addition to confirming the relationships between the TAM model, the study confirmed the relationships between the four NPT mechanisms and the TAM constructs by including the NPT constructs as external variables to the TAM model. Three of the NPT constructs – coherence, cognitive participation and reflexive monitoring – were found to relate positively and significantly with perceived usefulness and perceived ease of use. The levels of coherence and cognitive participation were high driven largely by the strong political will and government support. The eCHIS national rollout was officially launched by the current President of Kenya, a year after taking office, in an event that was widely publicised in the country. Hence, the huge support for eCHIS implementation, starting from the top leadership to the national and county governments, and multiple partners (55) could be seen as the driving force behind the CHWs’ high coherence and cognitive participation.

While fewer respondents alluded to existing written reports on the effects of eCHIS, its positive effects on fellow colleagues were evident in majority of the respondents. Majority of the respondents also stated that feedback was collected on eCHIS, which is then used to improve the functionality of eCHIS. This in turn related positively with the perceived ease of use of eCHIS. The importance of collecting and utilizing feedback on new digital technologies to improve not only the technologies but also the users’ way of working has also been highlighted in other studies (30, 56, 57).

The study found collective action to have the lowest average scores among the respondents driven largely by three measurement items related to provision of adequate resources, training, and management support. This finding corroborates other studies which also found collective action to have the lowest average score of all the constructs (58–61), with the items provision of adequate training, provision of adequate resources, and management support having the lowest ratings. The low scores on the three items highlight some implementation barriers and challenges facing eCHIS. Inadequate training of the CHWs on the eCHIS is one implementation challenge. The eCHIS trainings lasted on average 3 days, which were short especially for CHWs who had no prior experience with mHealth technologies, and for CHWs who are not technologically savvy. Adequate and regular training is crucial for the successful implementation of new DHTs among health workers.

Second, the study revealed insufficient management and technical support to facilitate the implementation of eCHIS. This is driven largely by the management structure of the community health system in Kenya. When community health providers encounter challenges with the eCHIS, they are required to first report them to their supervisors, the community health assistants. If the issues are easy to address, the CHAs would provide the support required. If the issues are complex, the CHAs would escalate them to the county government where the technical personnel would be required to handle them. However, discussions with the respondents during the data collection revealed that in most cases, technical support would come from the national government, and due to limited capacity to address the numerous technical challenges with the eCHIS nationally, the issues would take weeks and even months to be addressed. The study recommends hiring more technical personnel at the county and sub-county levels to ensure that technical issues facing the CHWs with the eCHIS are promptly and adequately addressed.

Similarly, inadequate financial resources emerged as an implementation barrier. The eCHIS in Kenya is funded by a consortium of partners including the national government, county governments and funders. Nonetheless, the funding is not equally distributed across the counties. For instance, the national government supports the remuneration of the CHPs with approximately Ksh. 2,500 (USD 19) every month for each CHP. The county governments are required to match this remuneration with a similar amount of Ksh. 2,500. However, reports indicate that the remuneration of CHPs by the county governments varies significantly, with some counties paying the CHPs the Ksh. 2,500, while others paying significantly more than that. Other CHPs receive no remuneration from their counties. This is also the situation with funders, where some counties receive significant financial support from the funders for the eCHIS implementation while other counties receive little to no support, depending on the interest of the funders (55). There is need for a streamlined and equitable resource allocation for the eCHIS initiative, ensuring that all CHWs are remunerated as per agreed guidelines, and that resources for eCHIS implementation are distributed fairly taking into account the resource needs of the counties.

The finding of no significant relationships between the two collective action items (provision of adequate training and management support) and perceived usefulness or perceived ease of use was contrary to the findings from other studies that assessed the role of training and management support in users’ acceptance of new technologies (8, 25, 62, 63). De Leeuw et al. (62) found that nursing management and leadership, as well as training on the job were facilitators of the respondents’ adoption of health information technology even though the respondents were considered digitally lagging. Korsah et al. (25) on the other hand found that management support in providing resources such as mobile phones, Internet, and financial incentives to the nurses facilitated their increased use of the mHealth technology in rural Ghana. The study by Charanthimath et al. (63) in rural India also stated that the respondents found the training they received was effective in equipping them with the skills and knowledge of how to use the POM app, particularly because the training was conducted in their local language. The training made the app easy to use for the respondents. On the other hand, the respondents in Arnaert et al.'s study in rural Burkina Faso (8) expressed the need for regular training and refresher courses to enable them to optimally use the mHealth technology to provide services to pregnant women. These studies show the importance of management support and training in adoption of digital health technologies.

While the study makes empirical contribution by using data to examine relationships between implementation process and acceptability of eCHIS, its theoretical contribution is equally significant. To the best of the authors’ knowledge, the study is the first to integrate the TAM model with an implementation science theory, the NPT. Additionally, the study contributes to the emerging literature applying the NPT questionnaire (the Normalisation MeAsure Development, NoMAD) particularly in LMIC contexts and in community health systems which have largely been excluded from the NPT literature. The study tested the reliability and validity of the NoMAD questionnaire and found that the questionnaire in its current form has reliability and validity issues particularly with respect to the collective action and reflexive monitoring constructs. Hence the theoretical constructs of NPT as measured quantitatively by the NoMAD questionnaire require additional testing and validation in different contexts and with different populations so as to enhance its reliability and validity.

### 4.1 Limitations of the study and suggestions for future research

One limitation of the study is that it used a purely quantitative research approach. Although the quantitative research design was adequate to address the study's objectives, the study would have benefited from deeper insights which can only be provided by qualitative methods. The study therefore recommends that future studies can adopt mixed methods research by incorporating both quantitative and qualitative designs. Qualitative research methods using in-depth interviews and focus group discussions with the community health workers would add deeper insights into the experiences of the CHWs with the eCHIS.

The second limitation of the study is that it limited the study population to community health workers only. There are many other stakeholders in the eCHIS ecosystem including the national and county governments, implementing partners, and community members who are impacted directly and indirectly with the use of the eCHIS. This study recommends that future studies should include the views of other stakeholders in the eCHIS ecosystem to better understand the positive and negative aspects of the eCHIS and the challenges facing its adoption.

Lastly, the study's respondents were drawn from only 5 counties out of the 47 counties of Kenya. This limits the generalizability of the study's findings to the CHWs population of Kenya. Future studies should consider a more inclusive sample to strengthen the generalizability of the findings. Additionally, future studies should also examine how the relationships between implementation process and acceptability of the eCHIS vary with the respondents’ socio-demographic characteristics including their gender, age, education level and geographical location.

## 5 Conclusions

The electronic community health information system in Kenya is widely accepted by the community health workers, and its implementation process can be considered successful. Nonetheless, a few implementation barriers including inadequate training, inadequate allocation of financial resources and technical support, as well as suboptimal management support need to be addressed to ensure that the eCHIS is fully adopted across the country. The application of implementation science in evaluating digital health technologies is a relatively new and emerging field. Implementation scientists acknowledge the complexity of digital health technologies which involve many moving parts. This study, situated in a developing country context and with low-cadre health workers, has demonstrated the significant role that implementation process plays in successful implementation of digital health technologies. The findings from the study are useful not only from theoretical perspective but also from practical and policy perspectives given the interest of many countries in implementing and scaling up digital technologies for healthcare workers. Countries planning national scale up of DHTs should ensure the training offered to the end users is adequate, taking into consideration the digital skills needs of the CHWs. They should also ensure that the resources (both financial and technical) are adequate and that management support for the end users is available during and after the rollout of the DHTs.

## Data Availability

The raw data supporting the conclusions of this article will be made available by the authors, without undue reservation.
